# Oxidized cellulose-based reaction mimicking thyroidal recurrence of disease: a case report and literature review

**DOI:** 10.1007/s12070-023-03556-0

**Published:** 2023-04-08

**Authors:** Carlotta Liberale, Erika Segato, Andrea Sacchetto, Marina Silvestrini, Roberto Saetti

**Affiliations:** 1 Unit of Otorhinolaryngology, Head & Neck Department, University of Verona, Piazzale L.A. Scuro, 10, Verona, VR 37134 Italy; 2 Department of Otorhinolaryngology, San Bortolo Hospital, Viale Ferdinando Rodolfi, 37, Vicenza, VI 36100 Italy

**Keywords:** Oxidized cellulose, Surgicel, Foreign body reaction, Cervical mass, Thyroidectomy

## Abstract

Oxidized cellulose is a hemostatic agent currently used in surgery. Foreign body reactions have been described after its use. This paper describes a foreign body reaction caused by oxidized cellulose in a young woman who underwent thyroid surgery. A patient underwent two thyroid surgeries for treatment of papillary carcinoma. Five years after the last surgery, an asymptomatic mass was detected with a routine ultrasound in the left thyroid cavity. After surgical removal, the mass turned out to be a foreign body granuloma based on oxidized cellulose sheets used in the previous surgery. The patient was discharged from the hospital without complications. The follow-up period was uneventful. Oxidized cellulose is a useful tool in surgery to control and prevent intra and post-operative bleeding. Even if it is a biodegradable material, sometimes it persists in the human body causing foreign body reactions that can be misdiagnosed.

**Supplementary Information:**

The online version contains supplementary material available at 10.1007/s12070-023-03556-0.

## Introduction

Oxidized cellulose is a polymer which composes different biomedical products, such as Surgicel® *(*Ethicon from Johnson & Johnson, New Jersey, US) or Tabotamp® (Ethicon from Johnson-Johnson, New Jersey, US). Oxidized cellulose is a hemostatic agent which is frequently placed in the bleeding site during surgery, to promote hemostasis [1]. The exact working mechanism of oxidized cellulose is unclear, but when the material saturates with blood, it turns into a gelatinous substance contributing to clot formation [2]. Oxidized cellulose is a biodegradable material that takes one to eight weeks to disappear from the human body [3]. Unfortunately, in some cases, oxidized cellulose doesn’t degrade and leads to foreign body formations, mimicking in most cases abscess or recurrence of disease.

This manuscript aims to describe the case of a patient who developed an oxidized cellulose-based foreign body reaction in the post-thyroidectomy bed several years after surgery. We also conducted a review of international literature to identify similar cases.

## Case Report

A 34-year-old female patient underwent right hemithyroidectomy in 2016 and then left hemithyroidectomy with selective neck dissection (level VI) for papillary thyroid carcinoma with one cervical lymph node metastasis. These surgical treatments were followed by adjuvant metabolic radiotherapy.

**Fig. 1:**
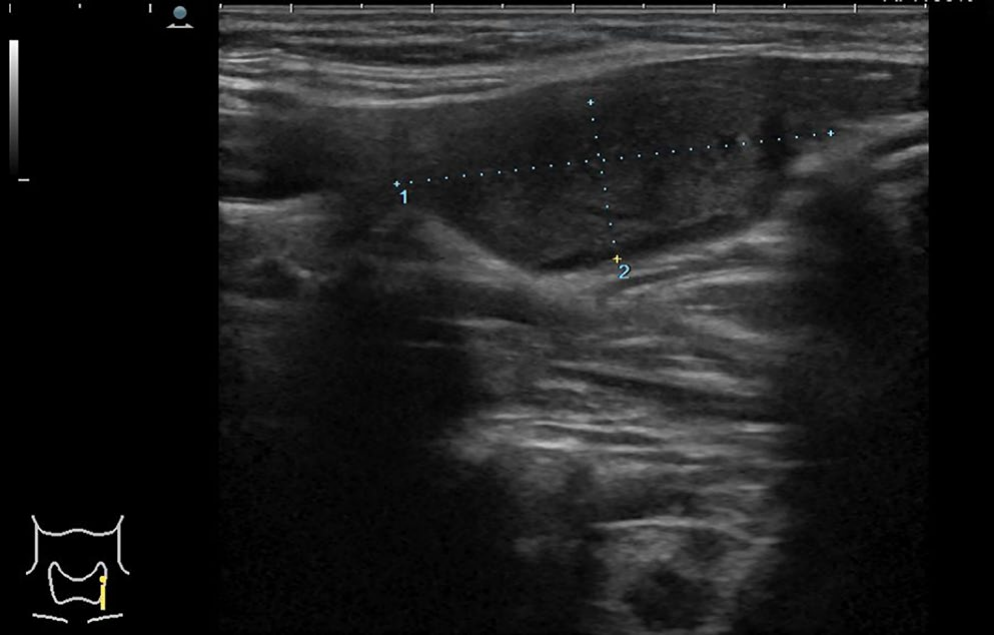
Ultrasound of the mass in the left post-thyroidectomy cavity

**Fig. 2:**
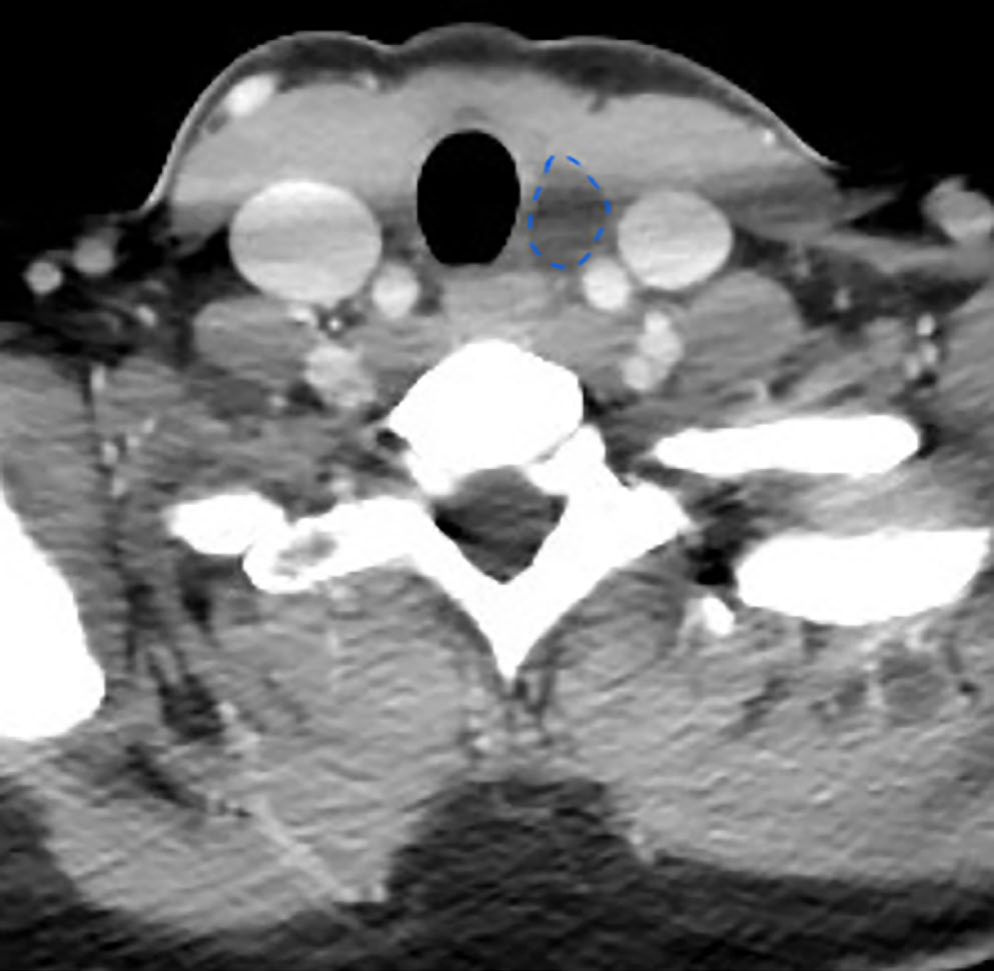
CT axial scan of the mass in the left post-thyroidectomy cavity. The dotted blue line surrounds the mass

Five years after left hemithyroidectomy, an asymptomatic mass was found in the left post-thyroidectomy cavity during a follow-up ultrasound (Fig. 1). The CT scan showed a hypodense mass with small intralesional calcifications (diameter 19 × 10 × 32 mm) (Fig. 2), without post-contrast enhancement. A fine needle aspiration cytology (FNAC) of the mass was performed and the cytological examination showed only amorphous material. Thyroid scintigraphy didn’t reveal any significant activity. Thyroglobulinemia has always been detectable with mild reaction to biological stimulus.

Considering the patient’s clinical history and the inconclusive clinical findings, surgical indication was given to exclude the disease’s relapse with certainty. The patient underwent surgery for the removal of the mass in the left post-thyroid cavity. During surgery, a capsulated mass was identified in the left thyroid lodge. The mass was delicately detached from the surrounding structures. A frozen section examination was performed and showed histiocytoid inflammation with giant cell reaction due to a foreign body.

**Fig. 3:**
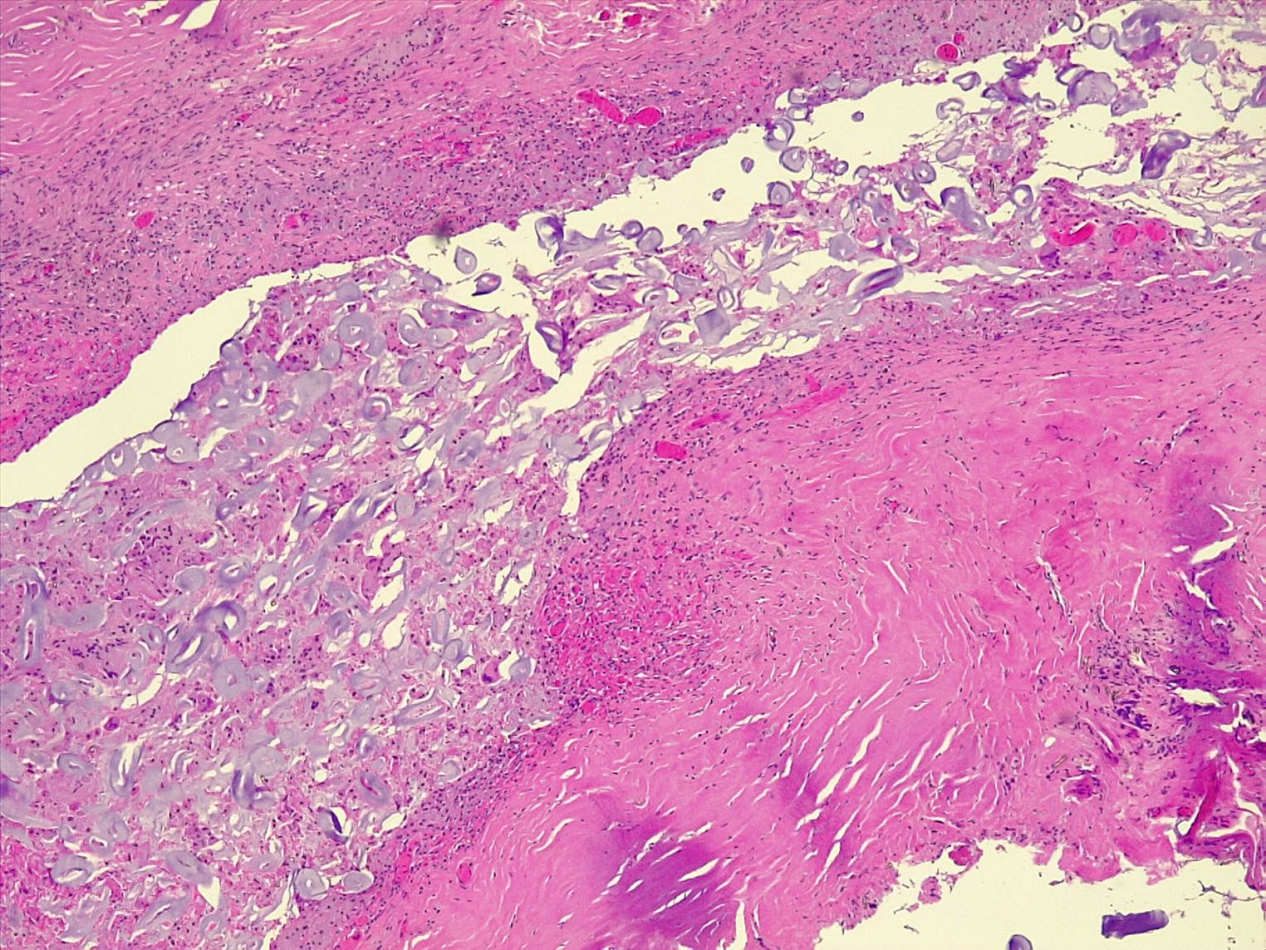
Histological examination: sections of fibrous tissue referable to cystic / pseudocystic wall, with histioid inflammation associated with giant cell reaction from a foreign body

The final histological examination confirmed the diagnosis (Fig. 3).

The patient was discharged from the hospital three days after surgery without complications. One month after surgery the patient was in good condition and the surgical wound was completely healed, without signs of inflammation.

Revision of the surgical reports confirmed the use of a Surgicel® sheet in the post-thyroidectomy bed during the previous surgery. This material caused the foreign body reaction and the progressive development of the neck mass.

## Literature Review

The research was performed using the PubMed database. The search string used was ((granuloma) OR (foreign body reaction) OR (abscess)) AND ((oxidized cellulose) OR (surgicel) OR (tabotamp) OR (gelita)). We included all the articles (111) found with the keywords, without limitation of time. Our last research was conducted in July 2022.

The inclusion and exclusion criteria of the research are summarized in Table 1.

**Table 1:** Inclusion and exclusion criteria

Inclusion criteria	Exclusion criteria
Head and neck district	Another district
English language	Other languages
Full text available	Full text or abstract unavailable

**Fig. 4:**
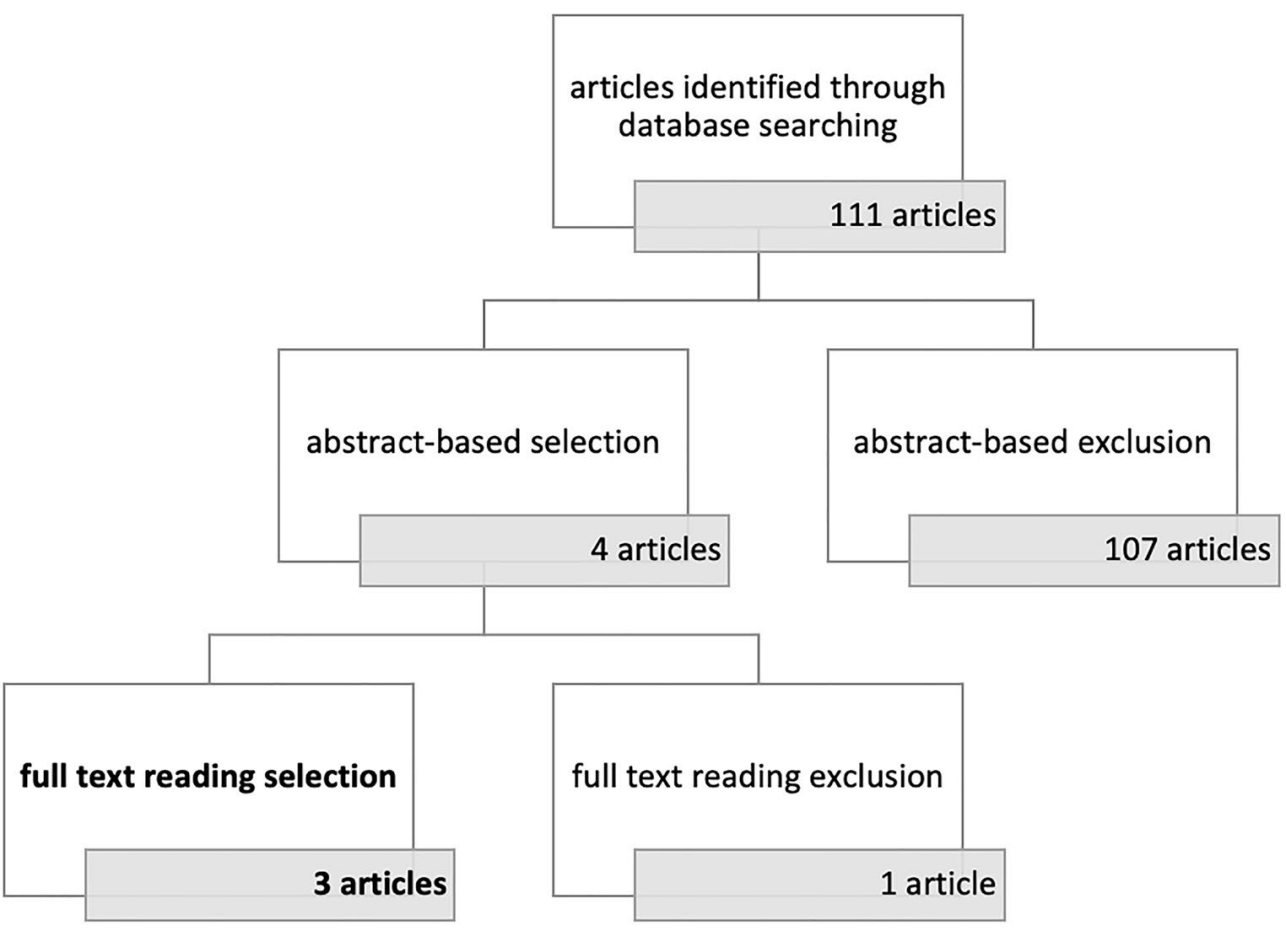
Paper selection process

At the end of the research, 111 articles were selected. Then, the results were filtered manually, choosing articles following the inclusion criteria. First, an abstract-based exclusion was made, with 4 articles left. After this, a further exclusion reading full-length papers was performed, with 3 articles selected. The paper selection process is shown in Fig. 4.

Two articles are case reports (*Azami Hassani FE et al. (4) and Royds J et al. (5)*) and one is a case series (*Hernández-Bonilla S et al. (6)*). From the case series, only patients with findings in the head and neck district were selected.

The oldest article was published in 2012 (*Royds J et al.*) and the most recent in 2021 (*Azami Hassani FE*).

The total number of patients with foreign body reaction due to oxidized cellulose in head and neck described in all articles was 6; 2 (33.3%) patients were male and 4 (66.7%) were females. The average age of the patients was 51.7 years (range 35–73 years).

All patients had surgery to treat their first pathology (4 thyroid surgery, 1 neck dissection and 1 surgery of temporomandibular joint). The average time between first surgery and the diagnosis of foreign body reaction was 12.3 months (range 1–24 months).

The site of foreign body reaction was the post-thyroidectomy bed in 4 patients, the laterocervical region and the temporomandibular joint in one patient each.

The results of literature review are summarized in Table 2.

**Table 2:** Results

Authors	Year	Study design	Patients	Gender	Age	First diagnosis	Treatment of first diagnosis	Time between first surgery and foreign body reaction	Localization
Royds J et al.	2012	Case report	1	F	56	Multinodular goitre	Surgery	1 month	Thyroid surgical bed
Hernández-Bonilla S et al.	2019	Case series	4	F	36	Papillary thyroid carcinoma	Surgery	24 months	Thyroid surgical bed
				F	49	Multinodular goitre	Surgery	13 months	Thyroid surgical bed
				M	35	Follicular adenoma	Surgery	18 months	Thyroid surgical bed
				M	73	Nasopharyngeal carcinoma	Surgery	15 months	Cervical lump
Azami Hassani FE et al.	2021	Case report	1	F	61	Chondrosarcoma of the temporomandibular joint	Surgery	3 months	Temporomandibular joint

## Discussion

The use of hemostatic agents is essential to prevent significant blood loss in surgical or emergency scenarios. Oxidized cellulose is an excellent biodegradable and biocompatible derivative of cellulose, which has become one of the most important hemostatic agents used in surgical procedures [4]. The biodegradation and biosorption of the oxidized cellulose in the surgical site have been reported to occur within several weeks [5, 6], in most of the cases it takes between one and eight weeks [3]. Nevertheless, Surgicel® has been reported as rarely mimicking pseudo-cancer, abscess formation and granuloma [7] in the head and neck surgical fields as well as in other anatomic regions.

Some Authors described cases of Surgicel® delayed absorption in the head and neck district; in particular *Azami Hassami et al.* [8] reported a case of suspected chondrosarcoma recurrence after Surgicel® use in the temporo-mandibular joint. The mass presented with a hypointense aspect at the MRI and its features were consistent with chondrosarcoma recurrence. The surgical approach was chosen to completely remove the mass; the histological examination showed residues of the hemostatic agent used three months earlier during the surgery.

*Hernández-Bonilla et al.* [9] reported some cases of pseudotumoral lesions after oxidized cellulose use. These lesions developed in the surgical bed after removal of different diseases: papillary carcinoma of the thyroid, follicular adenoma surgery, multinodular goiter and cervical lymph node excision for nasopharyngeal carcinoma. All patients underwent FNAC before surgery, which resulted in acellular, slightly laminated inorganic fragments surrounded by variable granulomatous inflammatory reactions. Similarly, in our case FNAC detected only abundant amorphous material with histiocytes. These cytological features are clearly not specific; however, the use of FNAC is always recommended in cases of suspected recurrence.

*Royds et al.* [7] reported a case of Surgicel®-based foreign body reaction post thyroidectomy 30 days after surgery, with abscess-like symptoms such as erythema and swelling of the neck. After surgical revision and Surgicel®’s removal, the patient’s symptoms resolved in two-three days.

In our case, the clinical evaluation of the patient, the preoperative FNAC of the lesion, US evaluation and CT scan of the neck could not allow to rule out the recurrence of papillary thyroid cancer. The radiological findings described a formation with uncertain diagnostic; however, its ultrasound features were consistent with residual thyroid parenchyma. During the surgery, the mass was described as well capsulated and attached to the prelaryngeal muscles in the left thyroid cavity. After minimal capsule rupture, a spillage of amorph-fibrous-like material was observed. The histologic examination ruled out potential cancer relapse, describing a pseudocystic lesion with histiocytoid flogosis and foreign body gigantocellular reaction.

Although very rare, the development of foreign body reaction to oxidized cellulose is a potential long-term complication in surgical procedures. In most of the described cases these lesions are completely asymptomatic and are individuated during routine follow up examination. The radiological and cytological findings are aspecific, thus the surgical removal of new onset masses seems to be the best option of treatment. The anatomopathological confirmation is mandatory to exclude potential recurrence of disease in oncological patients.

## Conclusion

Oxidized cellulose is a useful tool to control and perform safer hemostasis during surgery. Although it is a biodegradable material, some cases of foreign body reaction caused by its persistence are described. The development of these reactions can lead to the formation of masses in the surgical beds; the features of these lesions are often not distinctive, and a potential recurrence of disease must always be ruled out.

## Electronic Supplementary Material

Below is the link to the electronic supplementary material.

Supplementary Material 1

## Data Availability

Data sharing not applicable to this article as no datasets were generated or analysed during the current study.
