# Supplementary material for: Oxidized cellulose-based reaction mimicking thyroidal recurrence of disease: a case report and literature review
Source: Indian J Otolaryngol Head Neck Surg. 2023 Apr 8;75(3):2427–31. doi: 10.1007/s12070-023-03556-0 (PMC10447742; doi:10.1007/s12070-023-03556-0)
Supplement: Supplementary file 1 — Supplementary Material 1 [file 12070_2023_3556_MOESM1_ESM.docx]

COVER LETTER

Dear Managing Editor,

I am writing to submit our manuscript entitled “*Oxidized cellulose-based reaction mimicking thyroidal recurrence of disease: a case report and literature review*” for consideration, as a Head and Neck Pathology case report.

With this submission, we wanted to show an uncommon lesion we found during our practice at St. Bortolo Hospital in Vicenza.

During follow-up for an oncological thyroidal disease in a young patient, we found a mass with atypical imaging characteristics that was suspected of being a recurrence. Histological examination of the tissue after surgery confirmed a case of foreign body reaction, based on hemostatic agents used during a previous surgery.

Together with the case report, we conducted a review of the literature to find and analyze similar cases. We only found three articles that conformed to our inclusion criteria, with a single case similar to ours treating a thyroidal oncological disease.

This paper shows the importance of cautious use of hemostatic material when treating oncological disease, considering the possibility of their persistence in the body that can mislead surgeons and pathologists.

Each of the authors confirms that this manuscript has not been previously published and is not currently under consideration by any other journal. Additionally, all of the authors have approved the contents of this paper and have agreed to the Head and Neck Pathology’s submission policies. No statistical analysis was performed in the preparation of this manuscript.
